# Coherent Oscillations in a SrRuO_3_/BiFeO_3_ Superlattice

**DOI:** 10.3390/ma17061405

**Published:** 2024-03-19

**Authors:** Fardiman Ruli, Houssny Bouyanfif, Kyungwan Kim

**Affiliations:** 1 Department of Physics, Chungbuk National University, Cheongju 28644, Chungbuk, Republic of Korea; 2 Laboratoire de Physique de la Matière Condensée, Université de Picardie Jules Verne, 33 Rue Saint Leu, 80039 Amiens, France; houssny.bouyanfif@u-picardie.fr

**Keywords:** superlattices, coherent oscillations, zone-folding, magnetostriction

## Abstract

We investigated the ultrafast dynamics of a SrRuO_3_/BiFeO_3_ superlattice grown on a SrTiO_3_ substrate using a near infrared pump–probe technique at various temperatures. The superlattice exhibits a ferromagnetic order inherited from the SrRuO_3_ layer. The pump-induced changes in the reflectivity reveal periodic oscillations. We found that the oscillation frequency can be well explained by zone-folded acoustic phonon oscillations, whose dispersion depends on the sound velocity, density, and thickness within the supercell of each constituent layer. It is found that the observed oscillation frequency corresponds to the *A*_1_ mode, which suggests that oscillations are excited due to pump-induced expansion of the SrRuO_3_ layer that absorbs the pump photon. Temperature-dependent measurements reveal significant suppression of the oscillation amplitude in the ferromagnetic state. The suppressed amplitude is proportional to the square of the magnetization, *M*(*T*)^2^. This phenomenon can be attributed to a strong magnetostriction effect of SrRuO_3_ that suppresses lattice expansion upon pumping.

## 1. Introduction

Various functional materials with intriguing physical properties have been important ingredients for the development of current high-technology society. Materials with magnetic and/or electric order have been of particular interest for various applications, such as memory devices. In these days, more effort is being made to create and control those properties according to our own needs. For example, researchers have found new superconductors with higher transition temperatures by designing new materials with different chemical compositions, and are trying to utilize multiple functionalities by stacking materials with distinct but useful properties in the form of artificial heterostructures [1,2,3,4]. In particular, heterostructure engineering can provide us with a great degree of freedom in designing and controlling material properties. It is important to understand the physical properties of such new materials.

SrRuO_3_ (SRO) is a well-known itinerant ferromagnet with *T*_C_ = 160 K in bulk [5]. When it is grown in a thin film form, the transition temperature decreases to 150 K [6]. The transition temperature can decrease further in an ultrathin film, but the material shows magnetic and metallic properties down to thickness limit of a few unit cells [7,8,9]. This material is widely used as a ferromagnetic electrode as well as a buffer layer for various thin films of perovskite-based structures. An interesting spin structure of magnetic skyrmion has been observed when SRO is grown in a thin film heterostructure [10,11]. It is also found that electric and magnetic properties can be tuned by controlling a termination layer or a capping layer material [7,8]. All these properties make SRO one of the most popular materials for a constituent layer of artificial heterostructures seeking new functionalities as well as new and emerging phenomena.

BiFeO_3_ (BFO) is a multiferroic insulator, showing an antiferromagnetic order and also a ferroelectric order well above room temperature. A multiferroic material is of particular interest because its electric (magnetic) property can be controlled by the magnetic (electric) field due to strong electromagnetic coupling [12]. Although direct application of the antiferromagnetic property is limited, its strong electromagnetic coupling could provide an ideal platform in combination with other materials in the form of a heterostructure. BFO has a distorted perovskite rhombohedral structure in bulk but can form a tetragonal-like structure when it is grown in very thin film form under large compressive strain [13,14]. It is expected that tetragonal-like BFO will show a large spontaneous ferroelectric polarization value reaching up to 150 μC/cm^2^ [15].

When SRO and BFO form a superlattice, various new phenomena may arise. If SRO forms a superlattice with an insulating material, it is already known from many other cases that a metal-to-insulator transition would be observed as the number of unit cells of the SRO layer decreases [16,17]. It is also reported that the symmetry of the constituent material may also change depending on the relative thickness of each layer within the superlattice [3,4]. It is important to understand the general as well as unique properties of such superlattices.

The pump–probe technique is a powerful method that is becoming more popular in these days thanks to the development of ultrafast lasers. It includes not only the traditional near infrared (NIR) pump and NIR probe experiment but also various scattering and diffraction techniques utilizing more advanced short pulse sources, such as free electron bunches or femtosecond X-ray pulses from a free electron laser facility [18]. An ultrafast experiment allows us to investigate material properties in a nonequilibrium state. Pump excitation of a material via an ultrashort pulse can also drive the material into a completely different state that cannot be achieved by other controlled parameters in an equilibrium state such as temperature, pressure, or doping. A transient superconducting state would be one of the best examples that shows the uniqueness and interesting aspects of a transient state induced by an ultrashort light pulse.

Coherent oscillations are another phenomenon that does not have a counterpart in the equilibrium state. It also provides a unique opportunity to investigate changes in physical properties due to coherent phonon oscillations that are launched by pumping with a short pulse. Sudden excitation of the material can result in abrupt and coherent motion of ions over the volume excited by the pump. While thermal phonons are incoherent, and therefore, the average positions of atoms and the lattice structure do not change, coherently excited lattice vibrations can be considered to behave as if the material transiently has a different lattice structure during the vibration cycle. Such modification of the lattice structure is distinct from the lattice control achievable in an equilibrium state by applying external pressure that controls the lattice as a whole in a specific way. This phenomenon was the key for successful measurement of the deformation potential of Se displacement in FeSe [19], which was previously known only through theoretical estimation. As such, understanding the nature of the coherent oscillation can be very useful.

In this paper, we present the result of a NIR pump and NIR probe experiment on a [SRO13/BFO13]_10_ superlattice. In addition to the overall electronic signal, the transient reflectivity reveals coherent phonon oscillations, whose frequency corresponds to the zone-folded acoustic mode of *A*_1_ symmetry at the zone center. It is consistent with the displacive type of excitation of the coherent oscillation due to photon absorption by the SRO layer. Interestingly, the oscillation amplitude decreases with development of the magnetic order in the SRO layer. It can be understood in terms of a strong magnetostriction effect of SRO.

## 2. Materials and Methods

**Figure 1:**
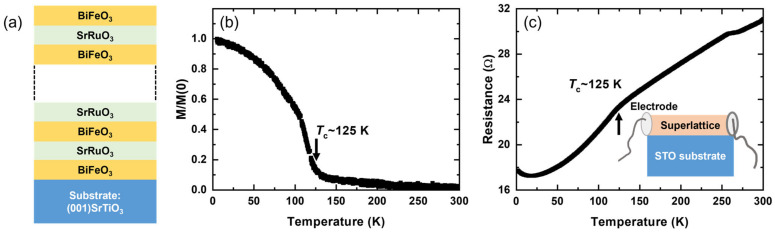
The [SRO13/BFO13]_10_ superlattice and its magnetization and transport properties. (**a**) Schematic diagram of the superlattice, (**b**) magnetization normalized by the lowest temperature value measured under 0.1 T using a vibrating sample magnetometer and (**c**) resistance measured with side contacts on the superlattice. The inset shows the side contacts used for transport measurement.

We grew a high-quality [SRO13/BFO13]_10_ superlattice on a (001) SrTiO_3_ substrate using the pulsed laser deposition method. The superlattice had 13 unit cells of SRO and 13 unit cells of BFO within one supercell period, which was repeated 10 times. An additional 13 unit cells of BFO were deposited as a capping layer, making the film symmetric, as schematically shown in Figure 1a. We confirmed that the in-plane lattice was fully strained from a reciprocal space map (not shown here). Because BFO is insulating, the transport property was investigated by making side contacts, as schematically depicted in the inset of Figure 1c. The magnetization was measured with a vibrating sample magnetometer under an applied magnetic field of 0.1 T over a wide temperature range from 5 K to 300 K.

The ultrafast dynamics were investigated by using the NIR pump–NIR probe technique in reflection geometry. The change in photoinduced reflectivity was measured employing a commercial Ti: sapphire amplifier laser system (Astrella, Coherent). The laser pulse had a center wavelength of 806 nm and a repetition rate of 5 kHz. The laser pulse width was 31 fs, as measured with an autocorrelator. The laser light was linearly polarized, and the pump and probe polarizations were perpendicular to each other. An additional polarizer was used before the detector to prevent pump photons scattered off the sample surface from entering the detector. The pump and probe beam shapes were measured by using a beam profiler, which showed Gaussian distributions with spot sizes of 93 μm and 53 μm in the full width at half maximum for pump and probe beams, respectively. The pump beam was incident at a near-normal angle on the sample surface and the angle of incidence of the probe beam was about 15 degrees. All measurements presented here were performed at pump fluence of 1 mJ/cm^2^. The change in photoinduced transient reflectivity was recorded as a function of the delay time at every 20 fs step between pump and probe pulses, controlled by using a linear optical delay line. A balanced detection technique with a reference beam split off from the pump beam was used to measure the tiny change induced by the pump. In addition, lock-in amplification with modulation using a mechanical chopper was adopted to further improve the signal-to-noise ratio. Time zero, at which the pump and probe pulses overlap each other, was found by measuring the second harmonic generation signal from a thin beta Barium Borate crystal and was also confirmed by measurement of where the roles of the pump and probe beams were exchanged. Temperature-dependent measurements of the change in transient reflectivity were performed in a wide temperature range from 20 K to 294 K using a cold finger-type closed cycle optical cryostat. 

## 3. Results and Discussion

### 3.1. Electric and Magnetic Properties

Figure 1 shows the magnetic and electrical properties of the superlattice sample. The magnetization confirms that the superlattice sample has a ferromagnetic order similar to SRO but with a reduced transition temperature of 125 K. The signature of the magnetic order can also be recognized in transport measurement by a kink that is marked with an arrow in Figure 1c. It is evident that there exists an additional anomaly in the resistance data around 260 K. It has been reported that subtle changes in the spin state of BFO, such as a spin reorientation transition, may arise between 10 K and 300 K [20,21,22]. Because BFO is insulating while SRO is metallic, the transport property should be dominated by the SRO layer. We suppose that interlayer coupling between the two materials resulted in the observed anomaly, whose origin is the spin state change in the BFO layer [22]. Slight resistivity upturn below 20 K may be attributed to the localization effect due to disorder [23]. In the following, we will focus our discussion on temperature-dependent evolution in the ultrafast transient reflectivity data across the ferromagnetic transition temperature.

### 3.2. Electronic Relaxation of Transient Reflectivity

**Figure 2:**
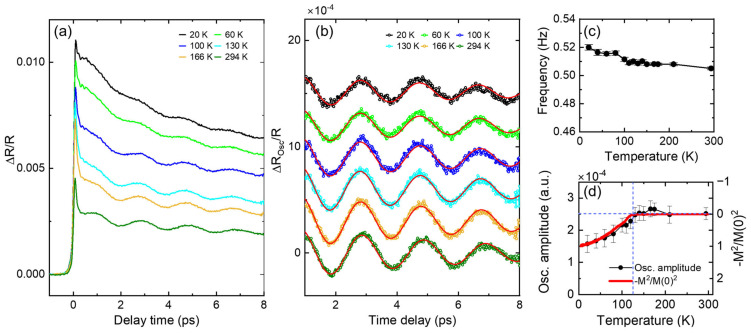
Transient reflectivity measured via the NIR pump and NIR probe method. (**a**) Temperature-dependent evolution of the pump-induced change in reflectivity. (**b**) Oscillatory part of the transient reflectivity after subtracting the electronic background using biexponential decay functions. The solid lines show damped oscillator model fit curves. (**c**) Fit parameter of the oscillation frequency and (**d**) oscillation amplitude at various temperatures together with the square of the magnetization, M(T)^2^.

Figure 2a shows the pump-induced reflectivity changes at various temperature between 20 K and room temperature. Because the SrTiO_3_ substrate is transparent in the NIR region, the strong pump beam reflected from the backside can result in a second pumping after about 8 ps, which corresponds to the round-trip time of the NIR pulse through the substrate. For the sake of simplicity, we analyze data up to 8 ps, as presented in Figure 2.

When the pump pulse arrives at the sample, the reflectivity increases abruptly, reflecting the change in the electronic state upon pumping. In the two-temperature model, the pump photon energy is considered to be deposited only into the electron system [24]. The photo-excited electron system is heated up immediately after pumping while the lattice still remains at the same temperature as in the equilibrium state. After quick thermalization of charge carriers, the electrons scatter, with the lattice-creating phonons approaching a quasi-equilibrium state between the electrons and the lattice. Because the reflectivity is dominated by the electronic state, the observed change in reflection is supposed to be dominated by electron temperature. While various relaxation processes play roles when the system returns to an equilibrium state in practice, electron–phonon scattering is still the dominant relaxation channel in a several-picosecond window.

One can notice that there is a small but fast decay component on a sub-ps time scale in the beginning, followed by a slower relaxation component lasting well beyond the time window shown in Figure 2a. As discussed in the previous paragraph, the reflectivity is dominated by the electronic state. Therefore, the observed response should be dominated by the change in the SRO layer, because the probe photon energy is smaller than the optical gap of BFO.

The amplitude and relaxation time of the fast and slow relaxation components show systematic variation depending on temperature. The most noticeable evolution is an increase in the slow component at lower temperature. We note that the slow component develops more with a ferromagnetic order below 125 K. This suggests that the relaxation dynamics of the slow component are strongly correlated with the spin state of SRO, although the component exists in the paramagnetic state as well. The physics related to these electronic responses will be discussed elsewhere after systematic measurements of additional SRO/BFO superlattices of controlled supercell thicknesses.

Recently, it has been proposed that electron–phonon coupling can be controlled by heterostructure engineering in the SrRuO_3_/SrTiO_3_ superlattice system [25]. The reported reflectivity change shows systematic evolution in the relaxation time as a function of the supercell thickness. While the transient reflectivity changes in both SRO-based superlattice systems are expected to be dominated by the SRO layer, the coexistence of the slow and fast components in Figure 1 is not observed in SrRuO_3_/SrTiO_3_ superlattices. Instead, the dynamics of the SrRuO_3_/SrTiO_3_ samples seem to have only the slow relaxation component of the SRO/BFO superlattice shown here. More systematic study is needed to understand the differences and similarities between these two superlattice systems.

### 3.3. Coherent Oscillations

In addition to overall relaxation of the electronic response, periodic oscillations were superimposed on all the measured data. Figure 2b shows the oscillatory signals obtained after subtracting the electronic responses from the raw data using biexponential decay function fits. The two exponentially decaying components correspond to the fast and slow relaxation components discussed in the previous section. The oscillation period is found to be about 2 ps. What is the origin of these oscillations? We note that the NIR pulse is absorbed only by the SRO layer because the optical gap of BFO is 2.67 eV, much larger than the NIR pump photon energy [26]. The frequency of about 0.5 THz is too small to be that of the optical phonons of SRO. These oscillations have not been observed in ultrafast experiments on SrRuO_3_ or BiFeO_3_ [27,28].

In pump–probe experiments, periodic oscillations can arise due to coherent lattice vibrations in the material. The generation of these coherent lattice vibrations is different from thermal phonon generation during the relaxation process of the hot electron system. Note that thermal phonons are incoherent with each other and cannot give rise to periodic modulations of reflectivity. Instead, coherent lattice vibrations are initiated at the moment of pumping over the pump-excited volume through either an impulsively stimulated Raman process or a displacive coherent phonon excitation process, due to the subtle change in the Coulomb force among ions caused by the absorption of the pump photons [29,30]. When the material is transparent at the pump photon energy, the impulsive Raman scattering process is the dominant process of coherent phonon generation. On the other hand, when the material absorbs the pump photons, the displacive excitation mechanism dominates over the stimulated Raman process. In the SRO/BFO superlattice, the SRO layer absorbs 1.55 eV pump photons. Therefore, we expect that the coherent oscillations are excited by the displacive mechanism. The observation of strong coherent phonon oscillations suggests that electron–phonon coupling in the superlattice is significant in the superlattice measured here.

**Figure 3:**
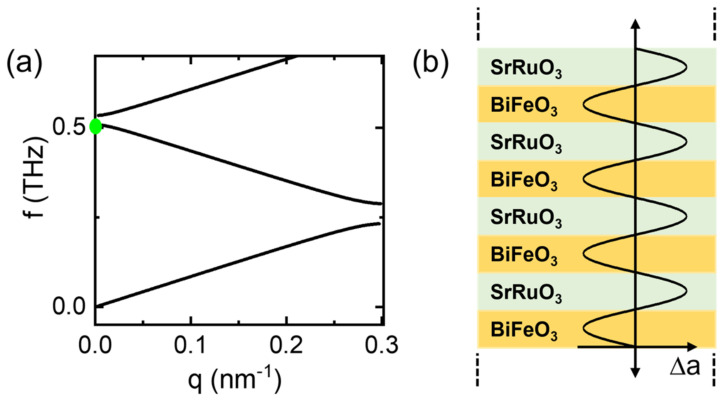
(**a**) Acoustic phonon dispersions folded into the reduced Brillouin zone of the superlattice and (**b**) lattice expansion in each layer of the phonon mode with *A*_1_ symmetry. The green dot in (**a**) marks the observed oscillation frequency at room temperature.

In superlattices, a new lattice period in the supercell reduces the Brillouin zone. Bragg scattering by the supercell results in folding of the electronic band dispersion and also phonon dispersion into the reduced zone. Such zone folding gives rise to additional modes at the zone center [31,32]. In superlattices, not only optic modes but also acoustic modes can become optically active due to the zone folding effect. The zone folding of the acoustic branch can be well described by using a layered elastic continuum model [33]. The new dispersion of a superlattice is written as

(1) $$\cos\left(kd_{SL}\right)=cos\left(\frac{\omega d_{SRO}}{v_{SRO}}\right)cos\left(\frac{\omega d_{BFO}}{v_{BFO}}\right)-\frac{1+\alpha^{2}}{2\alpha }sin\left(\frac{\omega d_{SRO}}{v_{SRO}}\right)sin\left(\frac{\omega d_{BFO}}{v_{BFO}}\right),$$

where *d* is the thickness and *v* is the sound velocity of each layer. The parameters *d_SL_* and *α* are defined as *d_SL_ = d_SRO_* + *d_BFO_* and α=vSROρSROvBFOρBFO, where *ρ* is the density of each layer. Figure 3a shows the zone-folded dispersion of the acoustic phonon from our superlattice sample obtained from Equation (1) using the values of *d_SRO_* = 5.16 nm, *d_BFO_* = 5.26 nm, *v_SRO_* = 6.3 nm/ps [34], *v_BFO_* = 4.76 nm/ps [27], *ρ_SRO_* = 6.489 g/cm^3^ [35], and *ρ_BFO_* = 8.34 g/cm^3^ [36]. We note that the sound velocity of BFO may vary depending on orientation and sample type. We employed the sound velocity observed in a thin film grown on a SrTiO_3_ substrate [27]. We expect that the sound velocity of a thin film grown on the same substrate should have the same sound velocity propagating in the depth direction perpendicular to the sample surface. The first-order folding of the acoustic mode to the Γ point results in two modes of *A*_1_ and *B*_2_ symmetry located at the lower and upper branches, respectively [33]. While the *A*_1_ symmetry mode has nodes of displacement in the middle of each layer, the *B*_2_ mode has nodes at the interfaces when the two constituent layers have the same thicknesses within one supercell. Because the α value is quite different from 1 in our superlattice case, there is a large gap between the frequencies of the two modes.

We further analyzed the oscillations by fitting a damped oscillator function to the oscillation component. We found that the oscillation frequency is about 0.505 THz at room temperature and increases to 0.52 THz at 20 K. An unusually large gap between the *A_1_* and *B*_2_ modes allows us to determine mode symmetry based on frequency. The observed frequency agrees well with the *A_1_* mode frequency expected according to Equation (1) within the error bar of the fitting result. The *A_1_* symmetry is consistent with the fact that only the SRO layer absorbs pump photons, which should result in instantaneous pump-induced expansion of the SRO layer corresponding to the displacive phonon generation mechanism. Because the BFO layer does not absorb pump photons, the BFO layer is expected to become compressed when the SRO layer expands, as illustrated in Figure 3b. We note that the *B*_2_ symmetry phonon can also be excited by the impulsive Raman scattering process. However, because of strong photon absorption by the SRO layer, the relative amplitude of the *A*_1_ phonon is expected to be much larger than that of the *B*_2_ symmetry mode generated via the Raman scattering process.

More interesting is the temperature-dependent evolution of the oscillation amplitude. As shown in Figure 2d, the oscillation amplitude decreases in a ferromagnetic state. The amount of temperature-dependent suppression of the oscillation amplitude perfectly matches the square of the magnetization value of the sample. How can we understand this behavior?

SRO is known to have a so-called invar effect [37]. That is, the volume does not become contracted as temperature decreases when it becomes ferromagnetic, in spite of anharmonic phonon–phonon interactions. In other words, SRO shows a very strong magnetostriction effect in the ferromagnetic state, canceling out the usual thermal contraction effect at lower temperatures. When a pump pulse excites electrons, the energy deposited into the system is known to suppress the magnetic order [28]. Therefore, while the excess energy given by the pump pulse tends to make the lattice expand due to the anharmonic phonon interactions, the suppressed magnetic order gives rise to the opposite effect, making the pump-induced expansion of the SRO layer become smaller. Because the observed phonon oscillations correspond to the *A_1_* mode, which is excited by the sudden expansion of the pump-excited SRO layer, the natural consequence is that the oscillation amplitude decreases due to the strong magnetostriction effect of SRO.

Interestingly, this magnetostriction effect is found to be proportional to *M*(*T*)^2^ of the sample, as shown in Figure 2d. A similar behavior has also been observed in SrRuO_3_/SrTiO_3_ superlattices [38]. The magnetostriction effect proportional to *M*(*T*)^2^ in the oscillation amplitude could be understood as follows. Usually, the coherent phonon oscillation amplitude is proportional to the pump fluence in a linear response manner. In other words, the oscillation amplitude increases as more energy is deposited to the system in most cases. However, in the ferromagnetic state of SRO, a portion of the pump photon energy is consumed to disrupt the magnetic order. The usual spin relaxation process happens via scattering with phonons, and it takes a much longer time, on the order of nanoseconds. However, it is well known that pumping a ferromagnetic material results in ultrafast demagnetization within a sub-picosecond time scale [39]. It is also suggested that the excess energy given by pump photons in SRO is preferentially used to suppress the magnetic order before heating the phonon system [28]. Therefore, part of the excess energy given by pump photons should be used to suppress the magnetic order and only the remaining part results in the usual lattice expansion. Because the magnetic energy in the ferromagnetic state should be proportional to *M*(*T*)^2^, the observed behavior in Figure 2d can be understood naturally. However, a direct relationship between transient magnetization and coherent phonon oscillations has not yet been investigated. Further study is desired to understand more details of the magnetostriction effect and its transient behavior in SRO and SRO-based superlattice systems. 

We note that the strong coupling between spin and observed acoustic phonon mode is manifested by a small but finite hardening of the phonon frequency in the ferromagnetic state, as shown in Figure 2c. That is, the phonon frequency increases more than the usual anharmonic hardening behavior as temperature decreases below *T*_C_. It is observed that spin–phonon coupling in Ca_2_RuO_4_ results in sudden softening of an optic phonon mode when the antiferromagnetic order develops [29]. Considering that the magnetic order is suppressed during the generation process of the phonon mode, the observed spin–phonon coupling must also be related to the strong magnetostriction effect of SRO. It will be interesting to investigate how coherent phonon oscillations behave in an extremely weak pumping limit such that the magnetic order still remains intact.

## 4. Conclusions

In conclusion, coherent acoustic phonon oscillations in the [SRO13/BFO13]_10_ superlattice were investigated using the NIR pump–NIR probe technique. The transient reflectivity exhibited periodic oscillations of 0.5 THz. Theoretical calculation of the layered elastic continuum model suggested that the observed phonon mode has *A_1_* symmetry, which has nodes of displacement in the middle of each layer. The excitation of the *A_1_* mode is attributed to a displacive-type generation mechanism resulting from pump-induced expansion of the SRO layer that absorbs the photon. Interestingly, the oscillation amplitude decreases in a ferromagnetic state, and this decrease is proportional to *M*(*T*)^2^. The reduced oscillation amplitude can be explained by the strong magnetostriction effect of the SRO layer. Our results demonstrate an intriguing example of emerging phenomena in superlattices, stemming from the artificial structure and unique properties of constituent materials. Further study is desired to understand the detailed relationship between coherent oscillations and transient magnetic order in SRO and SRO-based superlattices.

## Data Availability

The data that support the plots within this paper, and other findings of this study, are available from the corresponding author upon reasonable request.
